# The Institutional Worker

**Published:** 1913-07-05

**Authors:** 


THE
### INSTITUTIONAL WORKER
**Being a Special Supplement to "The Hospital"**

### OUR BUREAU OF INFORMATION.

### Rules for Correspondents.

1. Every letter must be accompanied by the coupon to be cut from the back cover (inside page) of The Hospital, current issue, and must contain the name and address of the correspondent with pseudonym for publication if desired. Replies by post cannot be given, save under exceptional circumstances at the Editor's discretion.

2. Letters from Approved Homes in reply to special needs published in the Bureau should state terms and full particulars, and be sent prepaid under cover to the Editor of the Bureau with name written across coupon for identification.

3. Proprietors of Homes which have not yet been entered on the List of Approved Homes, but have spare accommodation likely to suit special needs, are invited to write for an application form for registration. The fee for registration, which includes two announcements of the Home in the Bureau and other privileges, is 10s.

4. All communications to be addressed to the Editor of The Hospital, 28 Southampton Street, Strand, London, W.C., and marked "Bureau of Information."

### *THE INSTITUTIONAL OFFICER.*

Contributions from officers engaged in the work of institutions for the relief of the sick and afflicted, whether voluntary or rate supported, are invited, and will be paid for in accordance with scale if made use of.

---

#### Employment of Workhouse Inmates.

The question of the employment of the inmates of our workhouses is of a somewhat complex character, especially as regards the so-called able-bodied class. Sturdy vagabonds there are, unfortunately, in all workhouses, but their numbers are comparatively small; for in the Poor-Law sense able-bodied inmates are simply those between the ages of sixteen and sixty who are not in need of medical attention. Most of them are defective in body or mind, and their chances of obtaining permanent employment outside the workhouse are few and far between.

The really strong men fall into two divisions, the work-shy and the unfortunate. For the former no employment is too severe. The usual tasks given to them are stone-breaking, opium-picking, corn-grinding, or wood-sawing. The first two are rightly falling into disrepute. They are unremunerative in character and non-deterrent in effect. When once he has learned the trick of finding the grain of the stone, or of properly tearing the rope, the inmate can complete his task with comparative ease in considerably less than the allotted time. Workhouse masters would welcome more stringent regulations in dealing with these pests. To the man who is compelled by circumstances of illness or misfortune to seek shelter in th House, and who would willingly resume his work outside if possible, more leniency may be shown. Guardians usually try to help him by allowing him to leave the House for a time without his wife or children, in order that he may try to make a fresh start. His drawback usually is that he knows no trade, and finds a great difficulty in these days of keen competition in obtaining a fresh opening for his unskilled labour. In many workhouses the repairs to the building are carried out by indoor labour under the supervision of paid workmen, and the knowledge thus gained has enabled many of these men to gain a fresh footing outside.

The employment of the remaining male inmates consists mainly in the necessary cleansing of the building, work in the kitchen and bakehouse, gardening where land is available, and such easily learned tasks as brush and mat making. The female inmates are employed in the laundry and needleroom, and in scrubbing and cleaning.

The casual inmates are entirely separated from the inmates of the workhouse proper, and the work allotted to them is as far as possible of a deterrent character. Under the old conditions their treatment varied greatly in different casual wards, with the result that some were overcrowded, while others were rarely full. Since the Local Government Board has placed all the casual wards in the Metropolitan area under a separate authority (the Metropolitan Asylums Board), making the conditions equal in all, and steadily enforcing the task of work, the numbers have greatly diminished.—"J. H. C."

---

### *THE SICK AND DISTRESSED.*

**The Editor is prepared to make known without charge the needs of any who may be sick or in difficulties, and to guide them in making choice of Homes for treatment or convalescence. Reduced terms can often be arranged with Homes on the Approved List for those unable to pay full fees. Queries are specially invited from those who are engaged in any kind of philanthropical work. A new edition of the leaflet describing the purposes of the Bureau of Information is now ready and will be sent free of charge on application.**

#### Relief Required for a Distressing Case.

A very sad instance of a family in distress, owing to the illness of the breadwinner, has been brought to our notice, on whose behalf we appeal for assistance. The father contracted phthisis some time ago, and the progress of the disease has compelled him, after repeated breakdowns, to relinquish his business career. He has a wife and two little children dependent upon him; his private means are exhausted, and his income is limited to the sickness benefit of 10s. weekly to which he is entitled under the National Insurance Act, and a small allowance in kind, in lieu of sanatorium treatment which is not forthcoming. The mother prior to her marriage was an elementary school teacher, and she could, were she able to get work, support herself and her children by teaching. Meanwhile the family is in urgent need of help. There are several ways in which this might be rendered, as, for example, the provision of monetary assistance to tide over the present crisis, the reception of the father into a suitable Home where he could be cared for free of charge, or the employment of the mother in some suitable capacity whereby she may be enabled to support herself and her children. Co-operation can accomplish so much in a matter of this sort, and such help as our readers can give in one or other of the directions indicated will be gladly welcomed. The family is at present residing in the neighbourhood of Folkestone. Replies should be addressed "M. E. F.," 167b.

#### Societies for the Blind.

The Metropolitan Union of Institutions, Societies, and Agencies for the Blind was established in 1908. Its main object is to promote such intercourse and co-operation among existing institutions and agencies in London and the eight adjacent counties of Berks, Essex, Hants, Hertford, Kent, Middlesex, Surrey, and Sussex, as may lead to the co-ordination and extension of the work carried on. The Union has recently been strengthened by its incorporation under the Board of Trade. Not the least important of its many activities is the promotion of the prevention of blindness, by lectures and its co-operation with local education authorities and Guardians in seeing that all blind children of school age are being suitably educated and trained. There are seven similar Unions in the country, each of which sends a representative to the Union of Unions, so that the system of co-ordination of the numerous societies for the care and relief of the blind is very complete.—"Organiser."

#### Home Required for a Nurse.

A nurse who is unable to continue her work owing to weakness resulting from an illness contracted fourteen months ago is in need of a Home where she could rest for a month or two in bed, the treatment prescribed by her doctor. She is dependent upon her work for her living, and the most she can afford to pay is from 10s. to 12s. a week. Any of our Approved Homes able to accommodate this case are requested to reply to "Wayne," 166a.

### Home for Epileptic Required.

We shall be glad to hear from any home or institution, preferably near Halifax, able to receive a young woman suffering from epilepsy. A payment of 10s. weekly can be made toward her maintenance. Communications should be addressed "M. P.," 164a.

### Royal Sea-Bathing Hospital, Margate.

This institution does not receive convalescent cases as you have been led to believe. It is designed for the early treatment of most varieties of tuberculous diseases and certain other ailments that require, in addition to medical treatment, the benefits of sea air and sea-bathing. Cases of pulmonary tuberculosis are inadmissible, but an exception is made of children under fifteen years of age suffering from this disease in the early stages. Admission is by governor's letter and a monthly payment of £2 8s. for patients fifteen years of age and upwards, and £1 12s. if under that age. Cases are admitted without a governor's letter upon a monthly payment of £6. Application should be made to the Secretary, 13 Charing Cross, London, S.W.—"Ruth."

---

### *MISCELLANEOUS.*

### Sterilisation of Scalpels.

Whilst it is generally recognised that the sterilisation of metal instruments can be more reliably effected by boiling them than by the use of antiseptics, and accordingly most instruments are submitted to this process, a notable exception is made of cutting instruments such as scalpels, which of all instruments ought to be perfectly sterile. The objection to boiling these arises from the fear of impairing the keenness of the blades, and consequently many surgeons content themselves with the method of sterilisation by antiseptics. It is quite true that knives can be damaged in the course of boiling, both mechanically and through the softening of the steel, but it does not necessarily follow that they need be, if proper precautions be taken. Mechanical damage resulting from the contact of the knives with each other in the course of boiling can easily be avoided by placing them in a special rack in the steriliser. The more serious impairment of softening caused by the subjection of the knives to the temperature of boiling water for prolonged periods can be prevented by chilling them immediately upon their removal from the steriliser, by plunging them into cold alcohol, for it is the gradual cooling which destroys the temper of the steel. An instructive article dealing with this subject appeared in The Hospital on April 5.—"Theatre."

### The Odour of Iodoform.

The odour of iodoform may be removed from the hands by the application of mustard. Moisten the hands with cold water, place a small quantity of dry mustard in the palm, rub it well over the hands, and wash off with soap and water. The odour can be removed from utensils in the same way, with the exception that the mustard paste should be allowed to remain on for several hours; a solution of sodium hydroxide will also answer the purpose.—"N. Y."

---

### *INSTITUTIONAL FACTS AND FIGURES.*

### QUESTIONS FOR JULY.

**No. 1. (For the Officers of all Medical Institutions.)—State the character of your Institution, and describe your system of recording the admission, treatment, and discharge of patients admitted to the wards. Give descriptions of the various forms in use for the purpose, the method of dealing with them in the process of registration, and the method of filing history and treatment sheets. Add your further remarks.**

**No. 2. (For the Officers of Voluntary Hospitals.)—If you keep your accounts in accordance with the Uniform System, describe fully your method of checking, sorting, analysing, and filing your accounts for payment. Give such particulars as will clearly indicate the manner in which these are passed through your analysis journal, and onwards through th[illegible]. Add your further remarks.**

RULES.

The following rules must be observed:—

1. Contributions must be written on one side of the paper. They must bear the name and address of the sender and be accompanied by coupon to be cut from the back cover (inside page) of the current issue of The Hospital. A pseudonym must be chosen if the name is not to be published.
2. They must be addressed to the Editor of The Hospital, 28 & 29 Southampton Street, Strand, London, W.C., so as to reach him before the end of the month, and must be marked in the left-hand corner "Facts and Figures."

**A minimum payment of Half a Guinea will be made for each published answer.]**

---

### *EMPLOYMENT AND TRAINING.*

### How and Where to Train.

Domestic service is not usually considered a good preparation for those desiring to enter the nursing profession, and you will probably experience some difficulty in obtaining entry to a hospital as a nurse-probationer. You will find the fullest information upon the subject and a list of training schools in the book "How to Become a Nurse," published by The Scientific Press, Ltd., 28 and 29 Southampton Street, Strand, London, W.C., price 2s. 3d. post free.—"F. M. D."

It is somewhat difficult to obtain a general training after the age of thirty years, though at some training schools candidates are accepted up to the age of thirty-three, and in rarer instances thirty-five years. You will find a list of training schools and full particulars in the book referred to above.—"L. H."

There are many hospitals in London and the provinces which provide an excellent training; you cannot do better than study the book referred to above.—"H. L. McD."

### Nursing in Brazil.

The demand for British trained nurses in Brazil is very small. You might, however, apply to the British Hospital at Rio de Janeiro.—"M. B."

### District Nursing.

The possession of the C.M.B. certificate in the absence of a proper general training does not qualify you for district nursing. The Rural Midwives' Association could, we believe, give you some assistance in the direction you wish, and you might with advantage write to the Secretary at 47 Victoria Street, London, S.W.—"Fyfield."

### Cottage Hospital Furniture.

The book you require which will give you full information respecting the furniture and fittings of a small hospital is "The Cottage Hospital," by Sir Henry Burdett, published by The Scientific Press, Ltd., 28 and 29 Southampton Street, Strand, price 10s. 6d. The chapter entitled "Cottage Hospital Appliances and Fittings" deals with the special features you mention.—"H. McM."

---

### EDITOR'S LETTER-BOX.

*Case of Advanced Phthisis (M.E.F.).* We thank you for the further particulars respecting this case. We have published an appeal in these columns, and trust that it will be productive of some assistance. St. Michael's Home, Axbridge, receives advanced cases, and is free to members of the Church of England; if the patient comes within this denomination, you might meanwhile make application to the Sister-in-Charge of this Institution.

*H. L. Thurstans.*—We can only forward communications from our Approved Homes; we shall be pleased to consider an application from you for registration if you so desire.

*M.P.*—We should be glad if you will let us know whether you have been successful in finding a home for the case of epilepsy.

*Communications have been received from the following, and duly forwarded:*—Miss M. Day, Miss Warburton, Miss M. E. Gerrard, Miss C. Burton, and Mr. M. E. A. Martin.

**The Editor will be glad to receive correspondence and to consider contributions upon all subjects relating to institutional work, and affecting the welfare of institutional officers.**

### APPROVED HOMES ADDED TO THE LIST.—April to June 1913.

**Cornwall.**—5 Westwood, Liskeard. Principal: Miss Constance Burton, certificated nurse and masseuse. Medical, surgical, maternity, chronic, and convalescent cases received. Terms, from £2 2s.

**Essex.**—42 The Avenue, Highams Park, Chingford. Principal: Miss Hilda Wood (Medico-Psychological certificate). A comfortable Home for those suffering from mild mental and nervous diseases, and for chronic invalids. Terms, from 21s. a week.

**Essex.**—Tyler's Cross Farm, Roydon. Principal: Miss Hewitt. A Home for medical, surgical, and maternity cases, and delicate children. Special features: Rest-cure, Weir-Mitchell treatment, and massage. Terms, from two guineas weekly.

**Hunts.**—Berkley Villas, Eynesbury, St. Neots. Principal: Miss Rose E. Pearmain. A comfortable Home for invalids or convalescents. Terms, from 25s. weekly.

**London.**—136 Thornbury Road, Osterley Park, W. Principal: Miss R. G. Campbell, certificated nurse and masseuse. A new private Home for medical, surgical, and maternity cases, and for cases requiring massage and rest treatment. Accommodation also for delicate children, and for one case requiring open-air treatment. Terms, from £2 2s. to £6 6s.

**Provincial.**—Private Home for neurasthenic, slight mental, and chronic cases. Weir-Mitchell, massage, and electrical treatment. Under constant medical supervision by specialist. Terms, from £6 6s. a week. Applications should be made to "X. Y," THE HOSPITAL Bureau of Information.

### CHANGE OF ADDRESS.

Miss Edith M. Eastty, from Bracken Lodge, Marlowes, Hemel Hempstead, to Clovelly, Westcliff Park Drive, Westcliff-on-Sea.

### EDITOR'S NOTICES.

**Contributions:**

**Contributions should be written, or preferably typed, on one side of the paper only, and all articles sent in are accepted upon the distinct understanding that they are forwarded to THE HOSPITAL only.**

**The Editor cannot undertake to return MSS. not used, but when a stamped directed envelope is enclosed the MSS. may be returned if a special request is made.**

**Accepted articles and paragraphs of news will be paid for after publication at the scale rate.**

**Address:**

**To prevent delay all contributions and letters on editorial business must be addressed exclusively to the Editor, "The Hospital," 29 Southampton Street, Strand, London, W.C. It is important that this regulation shall be strictly observed.**

**Special Articles:**

**Special articles are invited, and questions, inquiries, and paragraphs upon all matters relating to the work, administration and management of general, special, mental, fever, cottage and convalescent hospitals, sanatoria, homes, institutions, societies and organisations for the treatment or care of the sick, injured and dependants of all classes. Every matter affecting the interests and welfare of the staff of all grades working in these institutions will receive special consideration.**

**A Bureau of Information:**

**We invite inquiries and applications for information and help in providing for that numerous class of sufferers who require special care or house-room which they cannot obtain in their own homes or secure for themselves. We cannot, however, prescribe, or recommend practitioners.**

### HANDBOOKS
### For TRAINED WORKERS

POCKET-GUIDE SP SERIES

**1/- net.** **1/1 Post free.**

**Bound in Limp Cloth.**
**Suitable Size for the Pocket.**

**The works which comprise the S.P. Pocket Guide Series are intended for ready reference, consequently the aim of the Publishers has been to make them as concise and lucid as possible. Each book is written by an expert on the subject of which it treats, and the utmost reliance therefore can be placed in the information it imparts.**

**Essentials of Fever Nursing.** By LYTTON MAITLAND, M.D. (Lond.), M.B., B.S., D.P.H. (Camb.).

**Manual and Atlas of Swedish Exercises.** (*With over 60 Illustrations.*) By THOMAS D. LUKE, M.D., F.R.C.S.

**Bandaging Made Easy.** (*Illustrated with over 90 Diagrams.*) By M. HOSKING, Sister-in-Charge, Tredegar House, Bow, E.

**How to Write and Read Prescriptions.** By LYTTON MAITLAND, M.D. (Lond.), M.B., B.S., D.P.H. (Camb.).

**Principal Drugs and their Uses.** By A PHARMACIST.

**Asepsis and How to Secure It.** By H. W. CARSON, F.R.C.S.

**Treatment after Operations.** *Rules for Nursing after General and Special Operations.* By MARY WILES.

**The Nurse's Duties before and during Operations.** By MARGARET FOX, Matron, Prince of Wales's General Hospital, Tottenham.

**Other works, in amplification of this Series, will be announced in due course.**

*Published by*

### The SCIENTIFIC PRESS, Ltd.

**28/29 Southampton Street, STRAND, LONDON, W.C.**

### GLOSSOP UNION.

CERTIFICATED NURSE.

